# Time-Restricted Feeding during Puberty Ameliorates Adiposity and Prevents Hepatic Steatosis in a Mouse Model of Childhood Obesity

**DOI:** 10.3390/nu13103579

**Published:** 2021-10-13

**Authors:** Francesc Ribas-Aulinas, Marcela Parra-Vargas, Marta Ramon-Krauel, Ruben Diaz, Carles Lerin, Trinitat Cambras, Josep C. Jimenez-Chillaron

**Affiliations:** 1 Institut de Recerca Sant Joan de Déu, Hospital Sant Joan de Déu, Esplugues, 08950 Barcelona, Spain; fribasaulinas@gmail.com (F.R.-A.); marcelacatalina.parra@sjd.es (M.P.-V.); marta.ramon@sjd.es (M.R.-K.); ruben.diaz@sjd.es (R.D.); carles.lerin@sjd.es (C.L.); 2 School of Medicine, University of Barcelona, 08036 Barcelona, Spain; 3 Department of Biochemistry and Physiology, School of Pharmacy, University of Barcelona, 08028 Barcelona, Spain; cambras@ub.edu

**Keywords:** time-restricted feeding, childhood obesity, small litter, non-alcoholic fatty liver disease, circadian rhythm, pubertal nutritional intervention

## Abstract

Background: Time restricted feeding (TRF) refers to dietary interventions in which food access is limited during a specific timeframe of the day. TRFs have proven useful in improving metabolic health in adult subjects with obesity. Their beneficial effects are mediated, in part, through modulating the circadian rhythm. Nevertheless, the translation of these dietary interventions onto obese/overweight children and adolescents remains uncharacterized. The objective of this study is to explore the feasibility of temporal dietary interventions for improving metabolic health in the context of childhood obesity. Methods: We have previously developed a mouse model of early adiposity (i.e., childhood obesity) through litter size reduction. Mice raised in small litters (SL) became obese as early as by two weeks of age, and as adults, they developed several obesity-related co-morbidities, including insulin resistance, glucose intolerance and hepatic steatosis. Here, we explored whether two independent short-term chrono-nutritional interventions might improve metabolic health in 1-month-old pre-pubertal SL mice. Both TRFs comprised 8 h feeding/14 h fasting. In the first one (TRF1) Control and SL mice had access to the diet for 8 h during the dark phase. In the second intervention (TRF2) food was available during the light:dark transitions. Results: TRF1 did not alter food intake nor ameliorate adiposity in SL-TRF1. In contrast, SL-TRF2 mice showed unintentional reduction of caloric intake, which was accompanied by reduced total body weight and adiposity. Strikingly, hepatic triglyceride content was completely normalized in SL-TRF1 and SL-TRF2 mice, when compared to the ad lib-fed SL mice. These effects were partially mediated by (i) clock-dependent signals, which might modulate the expression of *Pparg* or *Cpt1a*, and (ii) clock-independent signals, such as fasting itself, which could influence *Fasn* expression. Conclusions: Time-restricted feeding is an effective and feasible nutritional intervention to improve metabolic health, namely hepatic steatosis, in a model of childhood obesity. These data open new avenues for future safe and efficient chrono-nutritional interventions aimed to improve metabolic health in children with overweight/obesity.

## 1. Introduction

Childhood obesity is one of the most serious public health challenges of the 21st century. In 2016, around 41 million children under the age of five were overweight or obese. Childhood obesity is a major risk factor for other adult co-morbidities, including type-2 diabetes, cardiovascular disease, non-alcoholic fatty liver disease, kidney failure, and some types of cancer [1,2,3], which collectively shorten lifespan [4]. Current interventions are largely ineffective because their benefits are typically transient and tend to fade away over time [5,6]. The causes leading to childhood obesity are complex and include both genetic and environmental factors, such as high calorie intake and physical inactivity. In accordance, recent observational studies have established infant nutrition as a major factor that leads to rapid growth (i.e. weight gain), early adiposity and later disease risk [7]. Currently, the molecular mechanisms that contribute to the long-lasting metabolic effects associated to early nutrition and adiposity remain poorly characterized [8,9,10,11].

We have previously developed a mouse model of early adiposity (i.e., childhood obesity) through litter size reduction [12]. Mice bred in small litters (SL) exhibited rapid neonatal growth and developed obesity as early as by the age of 15 days [13,14]. As adults, SL mice displayed several metabolic disturbances, including glucose intolerance, insulin resistance, adult obesity and hepatic steatosis [13,14]. The liver played a major role in triggering early-onset insulin resistance. At the molecular level, hepatic steatosis and hepatic insulin resistance were attributed, in part, to misalignment of the hepatic circadian rhythm [15].

Time-restricted feeding (TRF) has recently emerged as a promising strategy for treating obesity and type-2 diabetes [16,17,18]. It is a dietary approach that limits the access to the food for a limited number of hours during the active phase of the day. Typically, the temporal feeding windows span from 6 to 10 h. Notably, the TRFs exert their beneficial effects, in part, through modulating the circadian rhythm [19], which in turn regulates metabolic outcomes [20]. However, to the best of our knowledge, there are no studies centered in children and/or adolescents [21,22]. Hence, it is currently unknown whether these types of chrono-nutrition interventions might be also effective for children with overweigh or obesity.

Here we aimed to explore whether TRF during the pre-pubertal period might improve the metabolism of SL mice. We show that 4-weeks intervention (8 h feeding/16 h fasting) ameliorated adiposity and prevented the development of hepatic steatosis. These effects were partly associated to better alignment of the hepatic circadian rhythm. Together, we provide evidence that early TRF, during pre-pubertal age, is an effective and safe means to improve metabolic health in the context of childhood obesity, without causing major impairments on appropriate growth.

## 2. Materials and Methods

### 2.1. Animal Care and Experimental Design 

**Figure 1:**
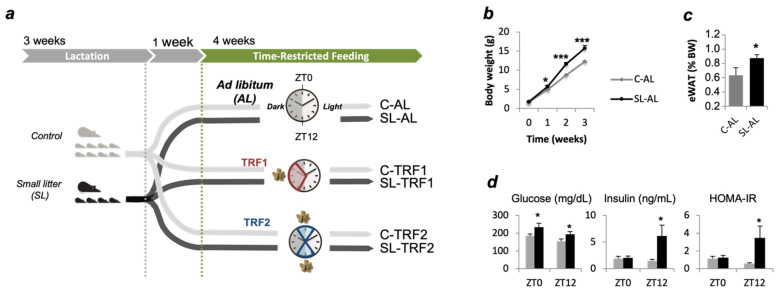
Litter size reduction induced overweight and adiposity in pre-pubertal mice. (**a**) Experimental design. Mouse model of early adiposity (i.e., childhood obesity) by litter size reduction. Litter size was adjusted at birth to eight pups (control group, C) or four pups per dam (small litter group, SL). At four weeks of age, C and SL mice were randomly assigned to 3 independent feeding groups: Ad libitum (AL), Time-restricted feeding procedure #1 (TRF1) and time-restricted feeding procedure #2 (TRF2). Therefore, we have now 6 groups: Control and SL mice fed ad libitum (C-AL; SL-AL), Control and SL mice maintained under the TRF1 procedure (C-TRF1; SL-TRF1) and C and SL mice maintained in the TRF2 protocol (C-TRF2; SL-TRF2). In both interventions, the mice had access to the diet for 8 h and fasted for 16 h. (**b**) Offspring growth trajectory during lactation in Control (*n* = 10) and SL (*n* = 10) mice. (**c**) Adiposity in pre-pubertal 1-month old Control and SL mice. (**d**) Plasma glucose and insulin levels in ad lib-fed C and SL mice, at the end of the 4-weeks intervention. Insulin sensitivity was assessed through HOMA-IR. All three measurements were taken at two independent times of the 24-h cycle, ZT0 and ZT12. Data represent means ± S.E.M. * *p* < 0.05 versus C-AL mice; *** *p* < 0.001 versus C-AL mice. Panel B, ANOVA; panel C, Student’s *t* test.

Protocols were approved by the Universitat de Barcelona Animal Care and Use Committee (CEEA-UB). ICR(CD1) mice (Envigo laboratories, Barcelona, Spain) were bred as previously described [16]. Briefly, at birth, litter size was adjusted to eight pups (control group, C) or four pups per dam (small litter group, SL) (Figure 1a). At weaning all mice had free access to standard chow (2014 Tekland Global, Envigo, Barcelona, Spain) until the beginning of the dietary intervention. Mice were maintained under constant temperature (21–23 °C), humidity (55 ± 10%) and dark-light cycles (12 h/12 h). In this study, metabolic analysis was conducted in males only, because as previously reported, SL females were protected against hepatic steatosis-hepatic insulin resistance [14].

### 2.2. Time-Restricted Feeding (TRF)

One-month-old C and SL mice were randomly assigned onto the ad libitum (AL) or the time-restricted feeding (TRF) groups. In total, we will study 4 independent groups: Control and SL mice fed ad libitum (C-AL; SL-AL), and C and SL mice that undergo dietary restriction (C-TRF; SL-TRF). Here we conducted two independent protocols, TRF1 and TRF2 (Figure 1a). Mice included in the TRF1 protocol had access to chow diet for 8 h per day during the dark cycle, from Zeitgeber time 14 (ZT14; 10:00 p.m.) to ZT22 (6:00 a.m.). ZT0 is lights-on (8 a.m.) and ZT12 is lights-off (8 p.m.) (Figure 1a). In the TRF2, mice had access to the chow during the light-to-dark transitions, from ZT10-ZT14 and ZT22-ZT2. Food access was controlled via programmable automatic feeders (SmartWaiter^®^, Cibertec, Madrid, Spain). Food intake was recorded at the beginning of the intervention, and every 48–72 h. Mice were euthanized, through inhalation of CO_2_, four weeks after commencing the dietary interventions. Liver was weighted, rapidly frozen in liquid Nitrogen, and stored at −80 °C for further analyses.

### 2.3. Liver TAG Analysis

Liver triglyceride content was determined as previously described [17]. Briefly, 100 mg of frozen liver sample was taken and homogenized in 500 μL of SDS 0.1% by Next Advance Bullet Blender Storm 24 (Next Advance, Troy, NY, USA). Then, 350 μL were taken and mixed with 350 μL of methanol. Equal volumes of chloroform were added; the hepatic lipids were extracted with methanol:chloroform and resuspended in ethanol, and the sample was chilled 30 min in ice. A volume of 48 μL of KCl 0.5 M was added and kept 30 min on ice. The samples were centrifuged (10 min at 2000 rpm at 4 °C), the SN discarded, and 300 μL of TAG (lower part) was left to evaporate O/N under the hood. The pellet was finally mixed in 50 μL of pure EtOH. Liver TAG concentration was determined through colorimetric methods (Free Glycerol Reagent #F6428, Triglyceride reagent #T2449; Sigma, Madrid, Spain). All samples were normalized by protein content using PierceTM BCATM (Thermo Scientific, Madrid, Spain).

### 2.4. Plasma Metabolites

Plasma insulin was determined in 10 μL of plasma by ELISA (Chrystal Chem, Zaandam, The Netherlands). Plasma triglycerides and cholesterol were measured using colorimetric-based enzymatic determinations in 5 μL of sample (COD11828 and COD1185, respectively; BioSystems, Barcelona, Spain). Whole blood glucose was measured with a Glucose Meter Elite (Menarini, Barcelona, Spain). 

### 2.5. HOMA-IR

Homeostasis model assessment of insulin resistance (HOMA-IR) was calculated according to the formula: plasma insulin (ng/mL) × plasma blood glucose (mg/dL)/405, as described elsewhere [23].

### 2.6. Real-Time Quantitative PCR (qPCR)

Total RNA was isolated from frozen tissue (Trizol, Sigma, Madrid, Spain) and used for cDNA synthesis (Promega, Madrid, Spain). Transcript levels were quantified by qPCR using the SYBR Green PCR Master Mix (Promega, Madrid Spain). Results were normalized to *b-Actin* and subsequently median-normalized to 1. The list of primers is detailed in the Supplementary Table S1.

### 2.7. Circadian Rhythm Analysis (Cosinor Method)

Liver RNA samples from C and SL mice were collected at different ZT time along the 24 h cycle. The presence of rhythmicity in each time series of data was statistically evaluated by adjusting data to a 24 h sinusoidal pattern and significance was tested using the cosinor method [24] and F test of sinusoidality [25].

### 2.8. Statistical Analysis

Pearson correlation analyses were performed with the statistical package JMP v14 (SAS Institute Inc., Cary, NC, USA). Results are expressed as mean ± SEM. Statistical analyses were performed using a two-tailed *t* test or a one-way ANOVA as indicated (IBM SPSS Statistics 19, Madrid, Spain). A * *p* value < 0.05 and *** *p* value < 0.001 was considered significant.

### 2.9. Data and Resource Availability

No datasets were generated or analyzed during the current study. Likewise, no applicable resources were generated during the current study.

## 3. Results

We have previously developed a mouse model of early adiposity (i.e., childhood obesity) through litter size reduction (Figure 1a) [12,13]. Mice reared in small litters grew faster that the controls (Figure 1b) and developed obesity by 4 weeks of age (Figure 1c). At the end of the 4-week intervention, the SL mice fed ad libitum (SL-AL) developed hyperglycemia, hyperinsulinemia, and insulin resistance (HOMA-IR), as determined at two independent periods of the light cycle, ZT0 and ZT12 (Figure 1d). In contrast, plasma triglyceride and cholesterol levels remained normal in SL-AL mice, when compared to the controls (Figure S1A). We tested whether two independent TRFs might effectively improve metabolism in these obese pre-pubertal mice (Figure 1a).

### 3.1. TRFs Normalized Insulin Sensitivity and Hepatic TAG Content in SL Mice

**Figure 2:**
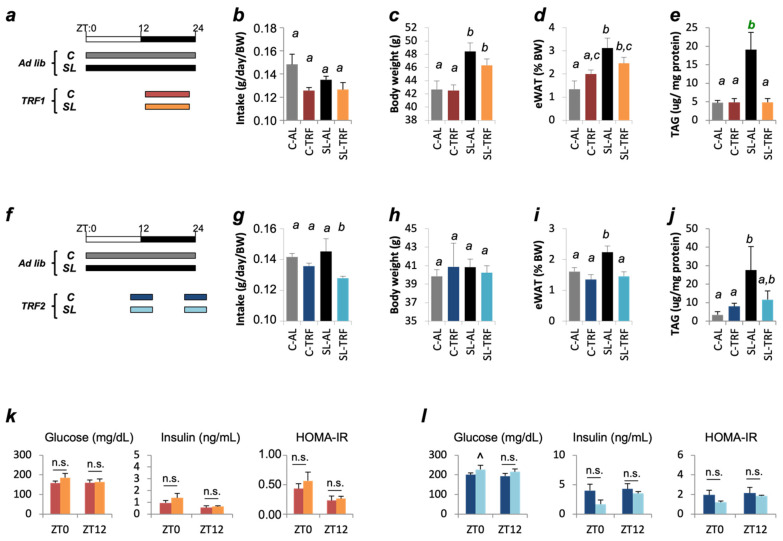
Two independent time restricted feeding (TRF) procedures ameliorated hepatic steatosis in small litter (SL) mice. (**a**) Scheme, representing the feeding-fasting distribution of C-TRF1 and SL-TRF1 mice during the day. The white bar represents the light cycle and the black one the dark cycle. ZT is the Zeitgeber Time. ZT0 corresponds to the beginning of the light cycle and ZT12, the beginning of the dark cycle. (**b**) Average food intake of C-AL (*n* = 10), SL-AL (*n* = 10), C-TRF1 (*n* = 5) and SL-TRF1 (*n* = 5) during the 4-week intervention period. (**c**) Body weight, (**d**) epididymal fat mass and (**e**) hepatic TAG content of C-AL, SL-AL, C-TRF1 and SL-TRF1 mice. (**f**) Scheme, representing the feeding-fasting distribution of C-TRF2 and SL-TRF2 mice across the 24-h cycle of one day. (**g**) Average food intake, (**h**) body weight, (**i**) epididymal fat mass and (**j**) hepatic TAG content of C-AL (*n* ≥ 5), SL-AL (*n* ≥ 5), C-TRF2 (*n* ≥ 5) and SL-TRF2 (*n* ≥ 5) mice. (**k**) Plasma glucose and insulin levels in C-TRF1 and SL-TRF1 mice, at the end of the 4-weeks intervention. Insulin sensitivity was assessed through Homeostasis Model Assessment of Insulin Resistance, HOMA-IR. (**l**) Plasma glucose and insulin levels, and (HOMA-IR) in C-TRF2 and SL-TRF2, at the end of the 4-weeks intervention. All three measurements were taken at two independent times of the 24-h cycle, ZT0 (*n* ≥ 5 both, C and SL) and ZT12 (*n* ≥ 5 both, C and SL). Data represent means ± S.E.M. ^ *p* < 0.1 versus Control mice, Student’s *t* Test; *a p* < 0.05 versus *b* or *c*, ANOVA; *b p* < 0.05 versus *a* or *c*, ANOVA; *c p* < 0.05 versus *a* or *b*, ANOVA. n.s. not significant.

In the first intervention (TRF1) (Figure 2a), food intake was similar among the four groups (Figure 2b). Body weight and epididymal fat mass remained comparable between SL-AL and SL-TRF1 mice (Figure 2c,d). Nevertheless, hepatic triglyceride (TAG) content was reduced in SL-TRF1 mice and reached similar levels than the control mice (Figure 2e). In the second intervention (Figure 2f) SL-TRF2 mice showed reduced unintentional food intake (Figure 2g). This effect was specific to the SL group because food intake remained unaltered in the C-TRF2 group. Reduced food intake was accompanied by improvements in adiposity, which reached similar values than the C-AL (Figure 2h,i). Hepatic TAG content was also nearly normalized in SL-TRF2 mice, when compared to the control groups (Figure 2j). Finally, both interventions normalized glucose and insulin content and reversed insulin resistance in SL-TRF1 and SL-TRF2 mice, when compared to their matched controls (Figure 2k,l). Neither TRF induced major modifications in plasma TAG and cholesterol content in SL mice (Figure S1B,C). 

**Figure 3:**
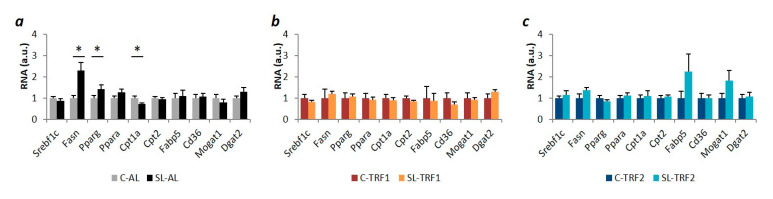
Time Restricted Feeding (TRF1) and (TRF2) restored the expression of genes involved in lipid synthesis and oxidation. (**a**) mRNA content (qPCR) of lipid-related genes in ad lib-fed (AL) mice (*n* ≥ 10 both, control (**c**) and small litter (SL)), TRF1 mice (*n* ≥ 5 both, C and SL) (**b**) and TRF2 mice (*n* ≥ 5 both, C and SL) (**c**). All samples were collected at the end of the 4-weeks intervention at ZT0. Data represent means ± S.E.M. * *p* < 0.05 versus C-AL mice.

We next measured the expression of genes involved in balancing TAG concentration, including de novo lipogenesis (*Srebf1c*, *Fasn*, *Pparg*), free fatty acid (FFA) oxidation (*Ppara*, *Cpt1a*, *Cpt2*), FFA transport (*Fabp5*, *Cd36*) and FFA esterification (*Mogat1*, *Dgat2*). In the SL-AL mice, increased TAG concentration might be attributed, in part, to enhanced lipogenesis (*Fasn*, *Pparg*) and reduced FFA oxidation (*Cpt1a*) (Figure 3a). Strikingly, both TRFs normalized the expression of *Fasn*, *Pparg* and *Cpt1a* in SL mice, suggesting that TAG normalization could be accomplished, in part, through blunting de novo lipogenesis and normalizing FFA oxidation (Figure 3b,c).

### 3.2. TRF-Associated Metabolic Improvements Are Partially Mediated by Clock-Dependents and Clock-Independent Signals

**Figure 4:**
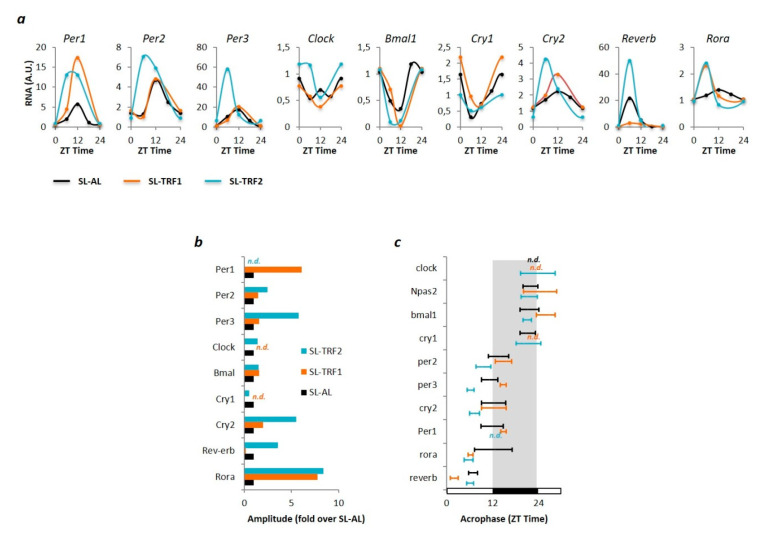
The chrono-nutritional interventions influence the expression of the hepatic clock genes in SL mice. (**a**) mRNA expression levels of the clock genes at different Zeitgeber Time (ZT) during a 24-h cycle in SL-AL mice (black) (*n* ≥ 10), SL-TRF1 mice (orange) (*n* ≥ 5) and SL-TRF2 (blue) (*n* ≥ 5) after 4 weeks of intervention. Gene expression levels were normalized to *b-Actin*. Amplitude (**b**) and Acrophase (**c**) of the clock genes during the 24 h cycle in SL-AL (black), SL-TRF1 (orange) and SL-TRF2 (blue) mice after the 4-week intervention. Note that Acrophase is showed in reference to the light (white) or dark (black) period of the day. The acrophase and/or amplitude for some genes could not be detected due to either lack of rhythmicity or low N value.

It is suggested that TRF-associated metabolic benefits are partly mediated through readjusting (peripheral) circadian rhythms [19]. Hence, we determined the hepatic expression of core clock genes, including *Per1-3*, *Cry1-2*, *Clock*, *Bmal1*, *Reverb*, and *Rora* in our mouse model. First, we found that the rhythmic expression of the clock genes was similar between young C-AL and SL-AL mice (Figure S2A). Hence, we focused our analysis on exploring whether the chrono-nutritional interventions modified patterns of rhythmic expression in the SL-TRF1 and SL-TRF2 groups, when compared to the obese SL-AL mice. As a result, we first found that the amplitudes of *Per1*, *Cry2* and *Rora* were higher in SL-TRF1 mice when compared to the SL-AL group (Figure 4a,b; Table S2). Likewise, both the amplitudes and acrophases of *Per1-3*, *Cry2*, *Reverb* and *Rora* were also modified in the liver of SL-TRF2 mice when compared to the SL-AL group (Figure 4a–c; Table S2). These data suggest that both TRFs actually induced significant re-adjustments in the hepatic circadian rhythm of the SL groups. It is worth noting that the clock genes that exhibited greater re-adjustments upon the dietary interventions, namely *Per1-3* and *Cry1*,*2* are actually involved in the regulation of hepatic lipid biosynthesis [26].

**Figure 5:**
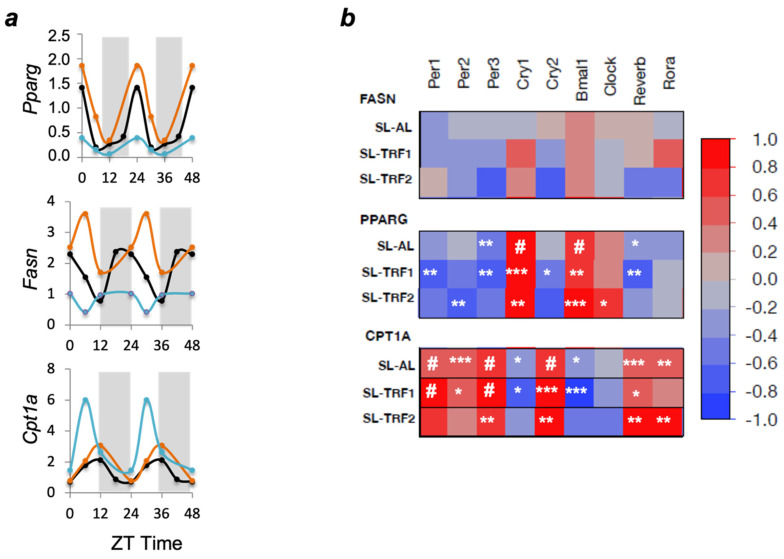
The dietary interventions modify the correlations between the clock genes and *Pparg-Cpt1a*. (**a**) mRNA expression levels of lipogenic genes (*Fasn*, *Pparg*) and FFA oxidation gene (*Cpt1a*) during a 24-h in SL-AL (black) (*n* ≥ 10), SL-TRF1 (orange) (*n* ≥ 5) and SL-TRF2 (blue) mice (*n* ≥ 5) at the end of the intervention. Data are presented as a “double-plot”, in which the 24-h expression is duplicated in order to better visualize the cyclic behavior of the genes. (**b**) Correlogram. The heat map indicates the Pearson correlation coefficients between the clock genes (columns) and lipid genes (rows). The red color designates positive correlations, whereas the blue color designates the negative correlations. The correlations that reached statistical significance are indicated as follows: * *p* < 0.05, ** *p* < 0.01, *** *p* < 0.001, # *p* < 0.0001, versus the SL-AL group.

Finally, we aimed to address whether the TRF-dependent changes in circadian rhythm might underlie the normalization of the previously identified lipid-associated genes, including *Pparg*, *Fasn* and *Cpt1a*. To this aim, we first determined whether *Pparg*, *Fasn* and *Cpt1a* exhibited rhythmic behavior and, second, whether the chrono-nutrition interventions further modified the pre-existing rhythmic patterns. We found that the three genes exhibited rhythmic behavior in SL-AL mice (Figure 5a). Importantly, both TRFs further changed their rhythmic behavior, albeit through different patterns: The TRF1 increased the amplitudes of the three genes, whereas the TRF2 blunted the amplitudes of *Fasn* and *Pparg*, and augmented *Cpt1a* (Figure 5a).

**Figure 6:**
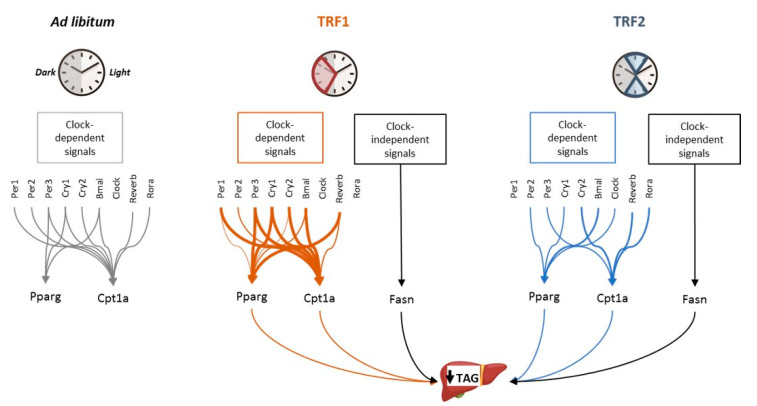
Working model-summary. The scheme represents the actual correlations established between clock genes and Fasn, Pparg and Cpt1a in SL mice (**left** panel). The TRF1 essentially strengthened the associations that already existed between the clock genes and Pparg-Cpt1a (**central** panel). This effect is typified with thicker arrows. The TRF2 introduced a new pattern of associations between the clock genes and Pparg-Cpt1a (**right** panel). Neither the TRF1 nor the TRF2 created any new significant association between the clock genes and Fasn. These data suggest that both interventions might contribute to reset hepatic TAG content through clock-dependent and clock-independent signals: Pparg and/or Cpt1a are re-set, in part, via clock genes, whereas Fasn is normalized through clock-independent mechanisms.

The key question now was to elucidate whether the clock genes might be involved in re-setting the cyclic patterns of *Pparg*, *Fasn* and *Cpt1a*. We partially addressed this issue through a regression analysis. First, in SL-AL mice the clock genes showed no significant correlations with *Fasn* (Figure 5b). In contrast, *Pparg* correlated with *Per3*, *Cry1*, *Bmal1* and *Reverb*, whereas *Cpt1a* strongly correlated with all clock genes except *Clock* itself (Figure 5b). The TRF1 tended to strengthen the already existing correlations with both *Cpt1a* and *Pparg*. Indeed, novel correlations were established between *Pparg* and *Per1*, *Cry2* (Figure 5b). Finally, *Fasn* did not significantly correlate with any clock gene in response to the TRF1. Together, these data suggest that the TRF1 reinforces the relationships that existed between the expression of clock genes and *Pparg*, *Cpt1a* in SL mice (Figure 6).

In the second intervention, the number and significance of correlations between the clock gens and *Pparg-Cpt1a* were reorganized as compared to the SL-AL mice (Figure 5b). For example, some correlations were lost with *Pparg* (*Per1*, *Per3*, *Cry2*, *Reverb*) and *Cpt1a* (*Per1*, *Per2*, *Cry1*, *Bmal1*), whereas a few others were newly established with *Pparg* (*Per2*, *Clock*). Again, *Fasn* did not show any correlation with the clock genes. These data suggest that the TRF2 re-shapes the relationships between the clock genes and the lipid-related genes *Pparg* and *Cpt1a* (Figure 6).

Although these associations do not provide causal relationships, they might suggest that restoration of hepatic TAG content could be attributed to (i) partial the re-alignment of the circadian rhythmicity (through *Cpt1a* and *Pparg*) and (ii) clock-independent signals, as pinpointed by the expression of Fasn (Figure 6).

## 4. Discussion

Chrono-nutrition strategies have become powerful tools for improving metabolic health in adults with obesity and/or diabetes [16]. Likewise, TRFs have been effectively applied to adult rodent models of metabolic disease [19]. However, the feasibility of TRF interventions in pre-pubertal subjects improving or preventing late-onset metabolic derangements is under-reported [21,22]. Here we provide strong evidence to support that short-term chrono-nutrition interventions have a profound beneficial impact on metabolism in pre-pubertal rodents with obesity. These data open the rationale for designing a similar type of interventions for children or adolescents with overweight/obesity.

In this study, we conducted two independent chrono-nutrition based interventions that consisted of cycles of 8-h feeding/16-h fasting. They differed regarding the time of the day when the feeding/fasting cycles were applied. In the first one (TRF1), food was available during to the dark cycle, from ZT14-ZT22 (Figure 1a). In the second one (TRF2), food was available during the dark:light transitions, from ZT10-ZT14 and ZT22-ZT2. In an attempt at translating them into the human practice, the TRF1 should be considered a “breakfast skipping” intervention, while the TRF2 should be a “lunch skipping” condition. 

Both interventions improved the metabolism of SL mice, although with slightly different final outcomes. These differences could be attributed, at least in part, to disparities in the feeding windows, which in turn might influence total calorie intake: In the TRF1, mice had access to the diet for 8 h during the dark cycle, which is the period of the day when rodents consume most of their calories. In the TRF2, this window was fragmented in two 4-h intervals that spanned a few hours of the light cycle, when rodents typically rest. We speculate that this fragmentation, together with conflicts with the light:dark signals, will shorten the effective time that SL-TRF2 mice spend on feeding and contribute to decreased food intake. Interestingly, this effect was specific to the SL-TRF2 mice. The Control healthy mice exposed to the same type of chrono-nutrition intervention did not experience reductions in caloric intake nor exhibited obvious metabolic derangements. These data suggest that these types of nutritional interventions are safe and could be potentially translated into the clinical practice. We recognize, though, that extreme cautiousness is needed before implementing these interventions in children. For example, “lunch skipping” interventions might be hard to implement in children. However, we argue that “breakfast skipping” approaches might be reasonably implemented in children. Firstly, it is shown that in adults, breakfast-skipping approaches are more efficient than lunch- or dinner-skipping formulas [27]. This is because “skipping breakfast” interventions have greater adherence to the treatment than other approaches. Secondly, a few studies have now suggested that age is an important factor in TRF, as young individuals may be more susceptible to its benefits than older counterparts. While clearly attractive, additional long-term experiments should be conducted in the future. A recent study reported some opposite data to ours: Pre-pubertal mice were subjected to short 4-week TRF intervention, followed by ad lib feeding for 4 additional weeks. This protocol compromised metabolic health, including obesity, hyperglycemia and hepatic steatosis in wild type mice [28]. While the authors have not explored whether their approach might have beneficial/detrimental effects in obese pre-pubertal mice, additional experiments are warranted to elucidate long-term effects of TRF in young rodents.

Our data support that in pre-pubertal obese mice, two independent short-term TRFs prevented the development of hepatic steatosis. This was accomplished, at least in part, through normalizing the expression of genes involved in lipogenesis (*Fasn*, *Pparg*) and FFA oxidation (*Cpt1a*). Some evidence supports that the TRFs improve metabolic health, at least in part, through modulating the circadian rhythm [29,30]. Indeed, we had previously shown that hepatic steatosis in SL mice might be attributed to dysregulation of peripheral clock genes [15]. Hence, here we speculate that normalization of TAG concentration in our model might be accomplished through re-setting the hepatic circadian rhythm. Indeed, we found that both TRFs induced dramatic changes in amplitude/acrophase of several core clock genes. Both interventions tended to increase the amplitudes of the core clock genes. In agreement, it is shown that other chrono-nutritional interventions also increased the amplitude of clock genes, which is generally associated to improvements in metabolic profile [31]. Interestingly, in our model the TRFs primarily modified those clock genes that are partially entrained by nutritional cues, including *Per1-3* or *Cry1-2* [20,32,33]. In turn, *Period* and *Cryptochrome* genes can regulate hepatic lipid content through directly influencing the expression of lipogenic genes [15,26]. Hence, here we proposed that the TRFs contribute to restore hepatic lipid content, in part, through re-setting the clock genes, which in turn, modify the expression of lipid-related genes.

We indirectly addressed this question through regression analyses (correlogram). While we recognize that it is not possible to establish causal relationships through regression assays, our data suggest that the hepatic clock genes might play a moderate role in modulating the expression of the downstream lipid-related genes *Pparg* and *Cpt1a*. Specifically, the TRF1 strengthened the correlations that already existed between the core clock genes and *Pparg-Cpt1a* (Figure 6). On the other hand, the TRF2 partially re-set these associations: Several new correlations were established whereas a few others vanished (Figure 6). Nevertheless, the fact that *Fasn* showed no direct correlation with either clock gene in any intervention strongly suggests that additional clock-independent mechanisms should contribute to normalize the lipid content in SL mice, [29,33] (Figure 6). For example, it has been recently shown that time-restricted feeding in pre-pubertal mice may permanently miss-program the gut microbiota composition [34,35] which in turn can influence whole body physiology, including hepatic steatosis [35]. Clearly, the potential impact of the gut microbiota and/or other fasting-derived signals in our model deserves being studied in the future.

## 5. Conclusions

In conclusion, time-restricted feeding during early life is a feasible nutritional strategy for preventing metabolic derangements in the context of childhood/adolescent overweight. Significantly, in our experimental setting, neither TRF induced obvious physiologic disturbances in the Control groups, which indicates that they are safe and, thus, potentially applicable for the pediatric population [11]. However, we recognize that this study has some limitations. Firstly, we have undertaken short-term experiments, and the long-term impact of these types of interventions will require further experiments before they might be implanted to humans. Secondly, the actual mechanisms involved in reversing hepatic steatosis and insulin resistance are far from being fully elucidated. Although we suggest that the clock genes might be involved in ameliorating hepatic steatosis, the data are based on associative studies. Therefore, additional experiments are needed to understand the molecular mechanisms involved in both processes. Finally, as we have extensively discussed, their implementation in clinical practice requires further careful evaluation. Specifically, here, we have conducted “breakfast skipping” and “lunch skipping” interventions. Pilot studies are required before translating them into the clinics. 

## Data Availability

No datasets were generated or analyzed during the current study. Likewise, no applicable resources were generated in this study.

## Supplementary Materials

The following are available online at https://www.mdpi.com/article/10.3390/nu13103579/s1. Figure S1: Plasma TAG and cholesterol levels were similar in SL-AL, SL-TRF1 and SL-TRF2 mice. Figure S2: The cyclic expression of clock genes was similar in C-AL and SL-AL mice. Table S1: List of primers for real-time quantitative PCR. Table S2: Summary of rhythmic parameters, including mesor, amplitude and acrophase.
