# Supplementary material for: Time-Restricted Feeding during Puberty Ameliorates Adiposity and Prevents Hepatic Steatosis in a Mouse Model of Childhood Obesity
Source: Nutrients. 2021 Oct 13;13(10):3579. doi: 10.3390/nu13103579 (PMC8538558; doi:10.3390/nu13103579)
Supplement: Supplementary file 1 [file nutrients-13-03579-s001.zip › nutrients-1391686-SI.pdf]

Figure S1.

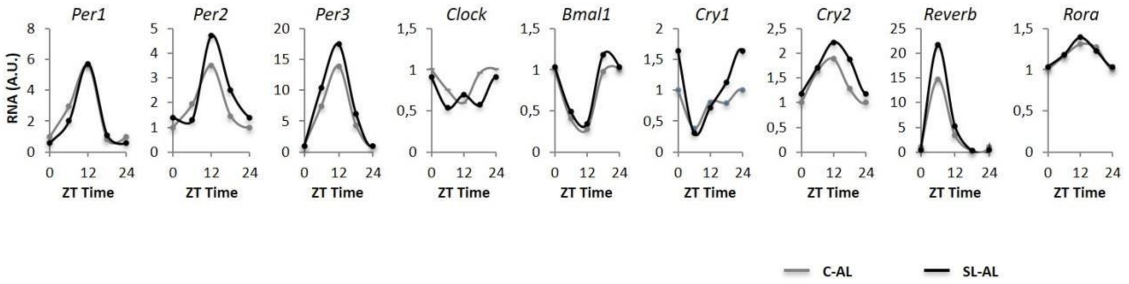

Figure S1. The cyclic expression of clock genes was similar in C-AL and SL-AL mice. mRNA expression levels of the clock genes during a 24-hour cycle in C-AL (grey) SL-AL (black) mice at the end of the experimental procedure. Gene expression levels were normalized to b-Actin.

Table S1. List of primers for real-time quantitative PCR.

Name	5' > 3' Sequence	Name	5' > 3' Sequence
**b-Actin-Fw**	CCACCGATCCACACAGAGTA	**Fasn-Fw**	GCTGCAAGCACAGCCTCTCT
**b-Actin-Rv**	AGTGTGACGTTGACATCCGT	**Fasn-Rv**	GGCATCATTGGGCACTCCTT
**Bmal1-Fw**	AACCATGTGCGAGTGCAGGCGC	**Mogat1-Fw**	GGTGAATGTTCCTGGGTGAG
**Bmal-Rv**	GGACTTCGCCTCTACCTGTTCA	**Mogat1-Rv**	CTGGTTCTGTTTCCCGTTGT
**Cd36-Fw**	CATTTGGCAGGTCTATCTACG	**Per1-Fw**	TGAAGCAAGACCGGGAGAG
**Cd36-Rv**	CAATGTCTAGCACACCATAAG	**Per1-Rv**	CACACACGCCGTCACATCA
**Clock-Fw**	GGAGGGAAAGTGCTCTGTTGTAG	**Per2-Fw**	GCGGAATCGAATGGGAGAAT
**Clock-Rv**	TTGCTCCACGGGAATCCTT	**Per2-Rv**	ATGCTCGCCATCCACAAGA
**Cpt1a-Fw**	GGCCTCTGTGGTACACGACAA	**Per3-Fw**	AGGTCTTCCCTGGCTTTGTT
**Cpt1a-Rv**	CTCAGTGGGAGCGACTCTTCA	**Per3-Rv**	ACAGCCCAATGTCTTCAGGT
**Cpt2-Fw**	CAGTGCACAGAAGCCTCTCTTG	**Ppara-Fw**	CTGGCATTTGTTCCGGTTCT
**Cpt2-Rv**	CTTCCCAATGCCGTTCTCAA	**Ppara-Rv**	TATTCGGCTGAAGCTGGTGTAC
**Cry1-Fw**	CTGTCCGCCATTGAGTTCTATG	**Pparg-Fw**	AGATCCGATCGCACTTCTCA
**Cry1-Rv**	CTGGCGTGGAAGTCATCGT	**Pparg-Rv**	GCCTCGGGCTTCCACTAC
**Cry2-Fw**	GTTTTTCAGGCCCACTCTACCTT	**Rev-erb-Fw**	GGTGCGCTTTGCATCGTT
**Cry2-Rv**	AGCCCAGGCCAAGAGGAA	**Rev-erb-Rv**	GGTTGTGCGGCTCAGGAA
**Dgat2-Fw1**	CTACTTCCGAGACTACTTTC	**Rora-Fw**	AGCTGCCACATCACCTCTCT
**Dgat2-Rv1**	CTGTGCTGAAGTTACAGAAG	**Rora-Rv**	TCCCCTACTGTTCCTTCACC
**Fabp5-Fw**	CAAAACCGAGAGCACAGTGA	**Srebf1c-Fw**	GGCCAGAGAAGCAGAAGAGA
**Fabp5-Rv**	CCCTCATTGCACCTTCTCAT	**Srebf1c-Rv**	CACATTTGAAGACATGCTCCAGCT

Table S2. Summary of rhythmic parameters, including mesor, amplitude and acrophase. P-values below 0.05 indicate that the gene has rhythmic behaviour along the 24-hours cycle.

		AL	TRF1	TRF2
Per1	Mesor	2.69 (1.95 - 3.43)	8.96 (8.14 - 9.78)	6,91
	Amplitude	2.46 (1.13 - 3.78)	11.67 (9.32 - 14.23)	---
	Acroph|time	11.96 (9.03 - 14.81)	14.95 (15.56 - 14.12)	---
	p	<0.001	<0.001	0,23
per2	Mesor	2.71 (2.2 - 3.22)	3.22 (2.6 - 3.84)	4.31 (3.41 - 5.21)
	Amplitude	1.84 (0.83 - 2.96)	2.73 (1.08 - 4.56)	4.49 (2.82 - 6.39)
	Acroph|time	13.99 (10.91 - 16.18)	15.59 (16.97 - 12.73)	9.28 (7.65 - 11.52)
	p	<0.001	<0.001	<0.001
per3	Mesor	8.95 (7.14 - 10.76)	10.66 (9.65 - 11.66)	9.48 (2.7 - 16.27)
	Amplitude	8.42 (5.16 - 11.77)	13.41 (10.58 - 16.52)	48.75 (28.88 - 68.69)
	Acroph|time	11.14 (9.13 - 13.38)	14.86 (15.52 - 13.95)	6.23 (5.35 - 7.23)
	p	<0.001	<0.001	<0.001
cry1	Mesor	1.05 (0.89 - 1.21)	1.4 (0.86 - 1.94)	0.81 (0.69 - 0.92)
	Amplitude	0.72 (0.38 - 1.1)	---	0.36 (0.1 - 0.66)
	Acroph|time	20.78 (19.16 - 23.13)	---	20.2 (18.12 - 24.66)
	p	<0.001	0,06	0,01
cry2	Mesor	1.72 (1.55 - 1.9)	2.27 (1.94 - 2.61)	1.44 (0.95 - 1.93)
	Amplitude	0.53 (0.22 - 0.85)	1.06 (0.5 - 1.82)	2.92 (1.48 - 4.39)
	Acroph|time	12.41 (9.09 - 15.4)	13 (15.59 - 9.07)	7.09 (6.05 - 8.61)
	p	<0.001	0,00	<0.001
reverb	Mesor	5.15 (3.06 - 7.24)	1.31 (0.93 - 1.7)	2,83
	Amplitude	13.21 (7.72 - 18.75)	1.77 (0.89 - 2.82)	46.97 (28.25 - 65.74)
	Acroph|time	6.82 (5.73 - 8.11)	8.12 (10.98 - 6.64)	6.14 (5.28 - 7.1)
	p	<0.001	0,00	<0.001
bmal1	Mesor	0.73 (0.61 - 0.85)	0.56 (0.41 - 0.71)	0.6 (0.52 - 0.69)
	Amplitude	0.46 (0.21 - 0.72)	0.72 (0.35 - 1.21)	0.7 (0.52 - 0.91)
	Acroph|time	21.18 (19.2 - 24.03)	2.8 (4.34 - 23.41)	20.86 (19.91 - 22.14)
	p	<0.001	0,00	<0.001
clock	Mesor	0.73 (0.59 - 0.86)	0.49 (0.4 - 0.59)	0.87 (0.72 - 1.01)
	Amplitude	---	---	0.31 (0.08 - 0.62)
	Acroph|time	---	---	23.81 (19.3 - 28.36)
	p	0,35	0,16	0,02
rora	Mesor	1.21 (1.12 - 1.31)	1.11 (0.96 - 1.25)	0.9 (0.65 - 1.15)
	Amplitude	0.18 (0.02 - 0.37)	1.4 (0.87 - 1.94)	1.51 (0.73 - 2.29)
	Acroph|time	12.6 (7.28 - 17.05)	6.19 (6.97 - 5.57)	5.85 (4.64 - 6.97)
	p	0,03	0,00	<0.001

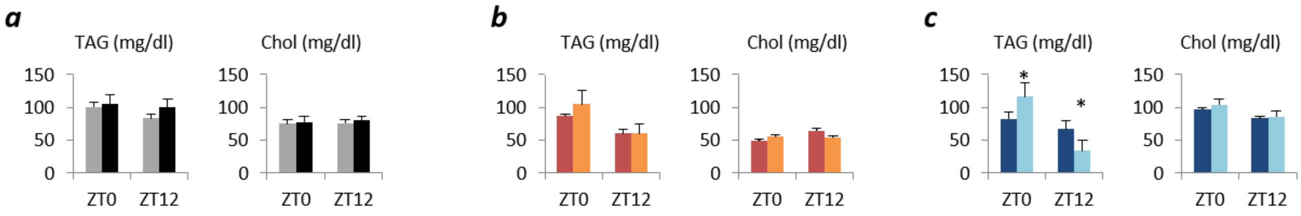

Figure S2. The cyclic expression of clock genes was similar in C-AL and SL-AL mice.
